# Supplementary material for: Effects of Salinity on Physiological, Biochemical and Gene Expression Parameters of Black Tiger Shrimp (Penaeus monodon): Potential for Farming in Low-Salinity Environments
Source: Biology (Basel). 2021 Nov 23;10(12):1220. doi: 10.3390/biology10121220 (PMC8698961; doi:10.3390/biology10121220)
Supplement: Supplementary file 1 [file biology-10-01220-s001.zip › biology-1415695-supplementary.pdf]

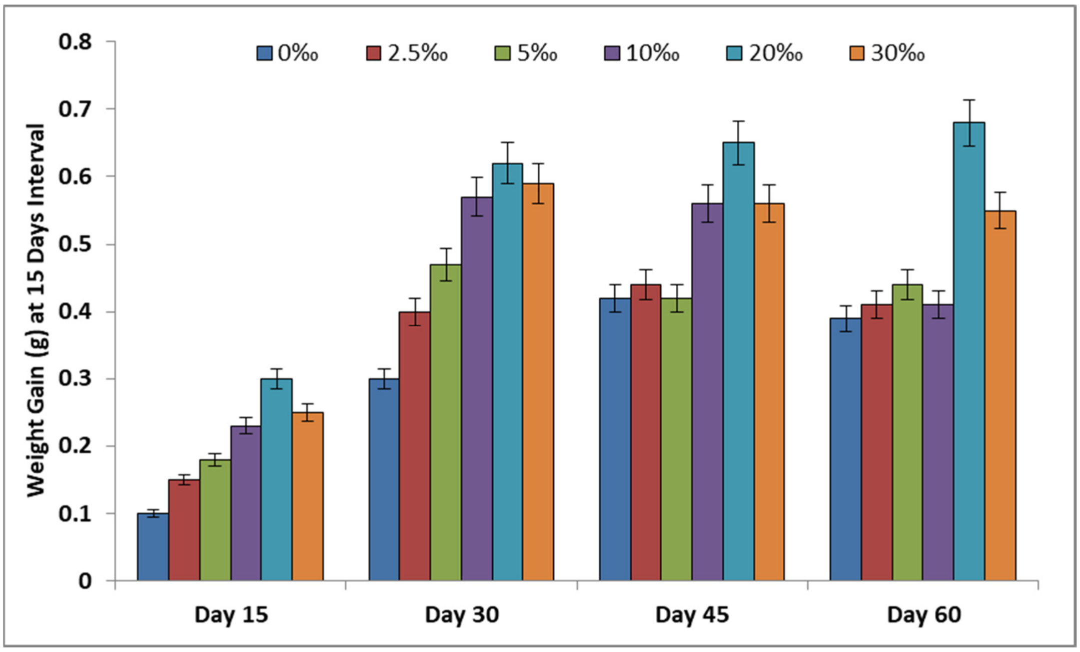

Figure S1: Weight gain of the experimental black tiger shrimp (*Penaeus monodon*) at every 15 days interval (mean ± S.D.) at six different salinity levels.

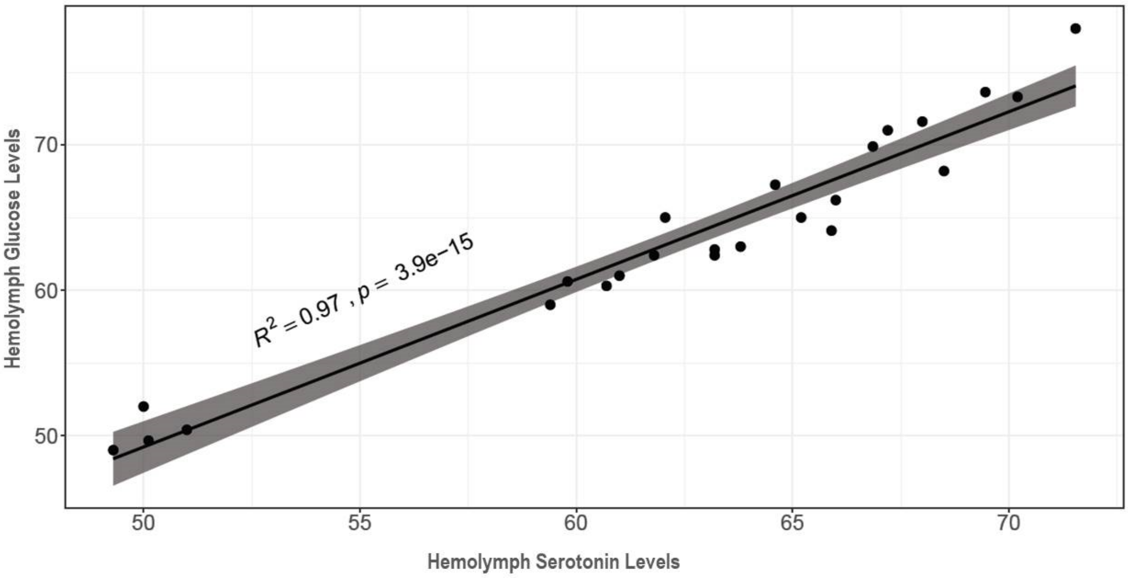

Figure S2: Correlation plot between hemolymph glucose (Y axis) and serotonin (X axis) levels of experimental *P. monodon* individuals at six different salinity levels.

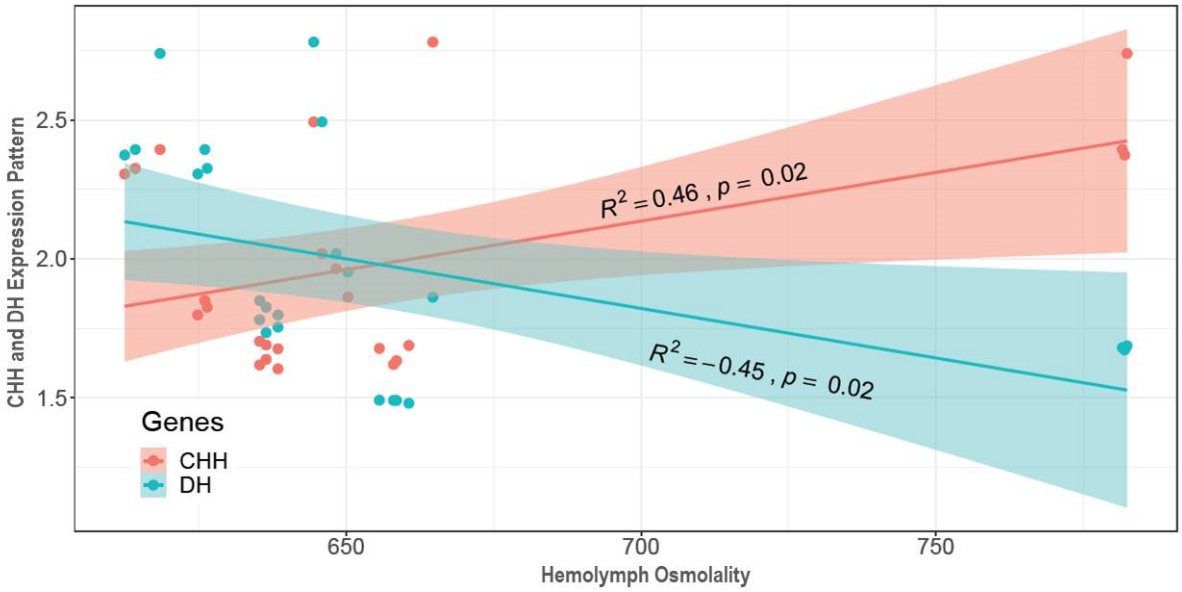

Figure S3: Correlation plot between hemolymph Osmolality (X axis) vs expression pattern of CHH and DH (Y axis).
